# Media portrayal of ethical and social issues in brain organoid research

**DOI:** 10.1186/s13010-022-00119-z

**Published:** 2022-04-13

**Authors:** Abigail Presley, Leigh Ann Samsa, Veljko Dubljević

**Affiliations:** 1 NC State University, Raleigh, NC USA; 2 UNC Chapel Hill, Chapel Hill, NC USA; 3 Department of Philosophy and Religious studies, NC State University, 101 Lampe Drive, Withers Hall 453, 27695 Raleigh, USA

**Keywords:** Brain organoid, Cerebroid, Bioethics, Neuroethics, Media

## Abstract

**Background:**

Human brain organoids are a valuable research tool for studying brain development, physiology, and pathology. Yet, a host of potential ethical concerns are inherent in their creation. There is a growing group of bioethicists who acknowledge the moral imperative to develop brain organoid technologies and call for caution in this research. Although a relatively new technology, brain organoids and their uses are already being discussed in media literature. Media literature informs the public and policymakers but has the potential for utopian or dystopian distortions. Thus, it is important to understand how this technology is portrayed to the public.

**Methods:**

To investigate how brain organoids are displayed to the public, we conducted a systematic review of media literature indexed in the Nexis Uni database from 2013–2019. News and media source articles passing exclusion criteria (*n* = 93) were scored to evaluate tone and relevant themes. Themes were validated with a pilot sample before being applied to the dataset. Thematic analysis assessed article tone, reported potential for the technology, and the scientific, social, and ethical contexts surrounding brain organoids research.

**Results:**

Brain organoid publications became more frequent from 2013 to 2019. We observed increases in positively and negatively toned articles, suggesting growing polarization. While many sources discuss realistic applications of brain organoids, others suggest treatment and cures beyond the scope of the current technology. This could work to overhype the technology and disillusion patients and families by offering false hope. In the ethical narrative we observe a preoccupation with issues such as development of artificial consciousness and “humanization” of organoid-animal chimeras. Issues of regulation, ownership, and accuracy of the organoid models are rarely discussed.

**Conclusions:**

Given the power that media have to inform or misinform the public, it is important this literature provides an accurate and balanced reflection of the therapeutic potential and associated ethical issues regarding brain organoid research. Our study suggests increasing polarization, coupled with misplaced and unfounded ethical concern. Given the inhibitory effects of public fear or disillusion on research funding, it is important media literature provides an accurate reflection of brain organoids.

**Supplementary Information:**

The online version contains supplementary material available at 10.1186/s13010-022-00119-z.

## Introduction

Growth of science and technology can lead to public inspiration or dread. Scientists can now coax stem cells in culture to grow into miniature tissues called *organoids*, which are designed to recapitulate organs in vitro, and developed from pluripotent stem cells to exhibit multi-cell differentiation and self-organization (Lancaster and Knoblich 2014). These small, complex 3D structures contain cells of the tissue of origin and are used to study development, physiology, and disease. The first big breakthrough in organoid biotechnologies was reported more than a decade ago in 2009, when Hans Clevers’ group created mouse intestinal epithelial organoids (Sato et al. (Sato et al. 2009)), shortly followed by human intestinal organoids (Sato et al. (Sato et al. 2011)). Since then, organoid research has expanded to recapitulate nearly all tissues (including lung, thyroid, liver, pancreas, etc.) in a wide range of species (Li and Belmonte (Li and Belmonte 2019)). As such, the biotechnology has carved out an important space in pre-clinical and basic science research pipelines. One rapidly developing area of organoid research is that of human brain organoids (sometimes called cerebral organoids, cerebroids, and embryoids, but for the purposes of this paper, we will refer to them as ‘human brain organoids’). Given the significant therapeutic and ethical implications raised by such research, brain organoids will be the focus of our investigation.

Human brain organoids are grown from induced pluripotent stem cells – normal cells that have been chemically coaxed into reversing into an embryonic state (Pasca et al. (Pasca et al. 2015)); human brains and human embryos are not harvested as tissue sources (Lancaster et al. (Lancaster et al. 2013)). While the brain organoids lack hallmark characteristics of higher-level cortical structures, they do grow to exhibit multiple neural cell types including neuroepithelial cells, astroglia, and distinct excitatory and inhibitory neurons, complete with synaptic connections (Chiaradia and Lancaster, (Chiaradia and Lancaster 2020)). As such, they provide researchers with a more complex and realistic model than just “cells in a dish.”

Brain organoid technology emerged from two distinct methodologies: guided and unguided. Guided development was first outlined by Sasai, who used reverse transcriptase PCR, growth factors and other chemical messengers to intentionally differentiate the organoids into desired brain regions (Qian, Song, and Ming (Qian et al. 2019); Sasai (Sasai 2013)). Alternatively, reverse transcriptase PCR can be used to direct differentiation of the organoid, using transcription factor expression to test for appropriate brain region development (Lancaster et al. (Lancaster et al. 2013)). Meanwhile, the Knoblich group took an unguided approach to brain organoid development, allowing the stem cells to self-differentiate and develop more spontaneously (Qian, Song, and Ming (Qian et al. 2019); Lancaster et al. (Lancaster et al. 2017)). As the organoids grow, researchers have noted electrical waves emitting from the organoids resembling those of a neonate, implying intercellular communication (Trujillo et al. (Trujillo et al. 2019)).

Lack of a vasculature limits both the maximum size of the organoid tissue and its faithfulness as a model of the brain. Three approaches have been developed to address this issue. Human-mouse chimeras are created by transplanting human brain organoids into the mouse brain (Chen et al. (Chen et al. 2019)). There, the endogenous vasculature penetrates and perfuses the grafted organoid (Mansour et al. (Mansour et al. 2018)). Human brain organoids have also been engineered to ectopically express vascular precursors that function to create a vasculature-like structure within the organoid (Cakir et al. (Cakir et al. 2019)). Finally, organ-on-chip approaches use microfluidics to mimic capillaries and enhance perfusion around organoid cultures (Park, Georgescu, and Huh (Park et al. 2019)).

Such development of brain organoid biotechnology has captivated the imagination of bioengineers, bioethicists, and the public alike given the unprecedented ability of these organoids to model complex neural circuitry and processes (Koo et al. (Koo et al. 2019)). As the name suggests, the organoids do not grow to be fully sized and functioning brains, but replicate certain brain *regions*, such as the hippocampus or parts of the forebrain (Lancaster et al. (Lancaster et al. 2013)). Importantly, the technology does not currently allow a comprehensive, functioning human brain to be grown in vitro. The forebrain, including the cerebral cortex and the hypothalamus, the midbrain, the hippocampus, and the cerebellum have all been successfully grown using brain organoid technology (Qian et al. (Qian et al. 2016); Qian et al. (Qian et al. 2018); Jo et al. (Jo et al. 2016); Sakaguchi et al. (Sakaguchi et al. 2015); Muguruma et al. (Muguruma et al. 2015)).

Inherent in the creation of brain organoids are a host of potential ethical concerns related to research, social and philosophical issues (see Table 1). While organoids are being touted as the future of clinical and physiological research, there is a growing group of bioethicists calling for caution in proceeding with brain organoid development (Koplin and Savulescu (Koplin and Savulescu 2019); Hyun, Scharf-Deering, and Lunshof (Hyun et al. 2020); Bredenoord, Clevers, and Knoblich (Bredenoord et al. 2017)). Given the many associated ethical issues with brain organoids (Hyun, Scharf-Deering, and Lunshof (Hyun et al. 2020)), it is necessary to briefly discuss the current ethical arguments for whether this model system should or should not be used as a research tool.

**Table 1:** Bioethical Issues in Brain Organoid Research

Type of Ethics	Description	Examples
Research (bioethics)	Issues encompassing the responsible conduct of research and relationship.	- Use of patient-derived tissues - Protection of research subjects
Social	Issues pertaining to the allocation of limited resources and the relationships of individuals and groups of people.	- Giving false hope for families struggling with dementia. - Setting priorities in research funding.
Philosophical	Issues pertaining to understanding the nature of the human experience	- Consciousness of brain organoids. - Moral status of brain organoids

### Claim 1: Brain organoids should be used because they reduce reliance on animal models

Currently, animal models are a prominent pre-clinical method for drug testing and general experimentation. However, Bentham’s consequentialist theory of animal ethics describes how animal experimentation presents ethical challenges. This theory posits that people should strive to achieve the “greatest benefit for the greatest number” i.e., maximizing benefits and happiness for all sentient creatures, while minimizing pain and suffering (Crimmins (Crimmins 2019)). In addition to ethical controversy, animal models also pose issues of reliability and accuracy (Johnson, Fenton, and Shriver, (Johnson 2020)). In contrast, species-specific human brain organoids offer an alternative to animal research while providing a modeling system that more precisely reflects the human brain. Marked differences in size, cytoarchitecture, genetic expression and cell dynamics between human and animal (especially mice) brains have posed great limitations to the neuropathologic and neurodevelopmental modeling potential of animals (Chiaradia and Lancaster, (Chiaradia and Lancaster 2020)). Brain organoids, while lacking the full complexity of a fully-formed human brain, are able to closely recapitulate discrete brain regions while exhibiting aspects of progenitor and mature neuronal populations, behaviors, and organization patterns characteristic of a human brain (Lancaster et al., (Lancaster et al. 2013)). In these ways, regional developmental patterns along with neural disease manifestations may be studied in a model highly reflective of the human brain in vivo. Brain organoids also facilitate the implementation of the widely accepted three Rs (replacement, reduction and refinement) principles for ethical research with animal models (Bredenoord, Clevers, and Knoblich (Bredenoord et al. 2017)). While it is unlikely that organoids will fully replace animal models in research contexts (Bredenoord, Clevers, and Knoblich (Bredenoord et al. 2017)), they may offer an ethical and reliable avenue complementary to established experimental methods to improve our understanding of neurodevelopment (Camp et al. (Camp et al. 2015); Simunovic and Brivanlou (Simunovic and Brivanlou 2017)) and disease mechanisms (e.g. the Zika virus (Qian et al. (Qian et al. 2017))), as well as test drug pathways (Koplin and Savulescu (Koplin and Savulescu 2019)). As such, some scientists are arguing for the introduction of a “comply or explain” paradigm: either researchers would use organoids in lieu of animals or explain why animal experimentation is needed (Bredenoord, Clevers, and Knoblich (Bredenoord et al. 2017)).

#### Counterclaim 1: Chimera brain organoids should not be used because they are too much of a moral gray area as mixed species constructs

Ethical arguments enter into a gray area when discussing human brain organoid implantation into animals, or chimeras. Chimeras are animals that contain human cells and can be anything from single-cell transplants to entire tissues (Chen et al., (Chen et al. 2019)). Brain organoid chimeras, then, possess some degree of human neural organoid tissue (Chen et al., (Chen et al. 2019)). Using animal models in the form of chimeras for research counters the argument that organoids can offer an alternative to animal experimentation, and raises questions as to the consequences for the animal. The conversation becomes one of both animal ethics and how the animal’s neural functioning is affected, as some are concerned it may become more “humanized” (Hyun, Scharf-Deering, and Lunshof (Hyun et al. 2020)). How, then, is the moral status of the chimera affected, and what is the potential for the development of human-like cognitive abilities (Yeager, (Yeager 2018))? It is thus difficult to assess how the potential benefits compare to the risks and where ethical lines should be drawn (Chen et al. (Chen et al. 2019)).

### Claim 2: Brain organoids should be used because they offer an ethical source of tissue to study human brains

Perhaps the most intuitive benefit of brain organoids is the potential to better understand the human brain itself, which could shed light on complex processes like memory, learning and attention. Such research is difficult to conduct and firm conclusions are rare, given the inherent ethical challenges associated with experimentation on human subjects and donor tissues. However, brain organoids bypass this issue, given their *in vitro* origin. An improved understanding of the human brain and cognition could have implications for improved characterization and treatment of disease (Fatehullah, Tan, and Barker (Fatehullah et al. 2016); Li and Belmonte (Li and Belmonte 2019)). These potential benefits are of such significance that some have claimed a “moral imperative” to continue development of brain organoids, given advances already made in diseases like the Zika virus, autism, and schizophrenia (Koplin and Savulescu (Koplin and Savulescu 2019)).

#### Counterclaim 2: Brain organoids should not be used because they may have or develop consciousness

There are a number of limitations attached to the application of brain organoids, which remain a source of controversy. One dominating concern is whether the brain organoids possess or can develop a form of artificially created consciousness (Koplin and Savulescu (Koplin and Savulescu 2019)). If this is the case, what then becomes the moral status of these brain organoids? Such issues are difficult to address, as they not only pose issues of regulation, but in measuring such abstract concepts as consciousness. Many researchers dismiss these concerns as unrealistic, insisting that brain organoid technology is not yet advanced enough to create fully sentient beings (Hyun, Scharf-Deering, and Lunshof (Hyun et al. 2020)). Some researchers, for example, instead cite more pressing concerns of developments of perception, particularly pain perception, among brain organoids (Yeager, (Yeager 2018)). In any case, while they are currently far from what most would consider a fully functioning brain or embryo, future advancement of brain organoid technology could yield more ethically concerning results (Hyun, Scharf-Deering, and Lunshof (Hyun et al. 2020)).

Though a recent and still developing technology, brain organoids and their potential uses are already being discussed in media literature, which refers to texts created for general public consumption. As such, media literature is designed to provide information to a lay audience, i.e., those with little to no background knowledge or experience on the given topic. Considering the complex and ethically involved issue of human brain organoid research, it is important to understand how this technology is being portrayed to the public.

How brain organoids are presented to the public can have far-reaching effects on public perception of the technology, which in turn may influence policy development, regulation and enforcement, as well as research funding. False hope leads to disappointment, which can strengthen the backlash surrounding dystopian fears about the technology. Alternatively, widespread public fear not reflected in current research may then challenge the future developments by starving it of public funds, which would give rise to a situation similar to some forms of stem cell research in the United States. Namely, throughout its initial stages of development, public fear and backlash directed U.S. policies to heavily restrict stem cell research, working to grind developments to a halt (Vakili et al. (Vakili et al. 2015)). Though these policies were later reversed, the time lapse initially worked to put the country behind in the stem cell research and publications, posing a barrier to publications and clinical advancements. In an effort to avoid a similar development in organoid research, which has significant clinical relevance, it is important that the public receives a comprehensive and scientifically grounded understanding of brain organoids, such that the technology is able to advance in a safe and ethically secure manner.

To better understand how this information is being conveyed to the general public, we studied how brain organoids technology is portrayed in media literature. Our investigation seeks to understand what promises are presented surrounding brain organoids research, how these promises are contextualized by current brain organoid research, and where this conversation is situated amongst relevant ethical and social issues. A previous study by Dubljević, Saigle, and Racine ((Dubljević et al. 2014)) explored media portrayal of transcranial direct current stimulation to reveal a previously unidentified disconnect between media coverage and scientific research as well as a lack of regulatory clarity. In a similar approach, here we conducted qualitative analyses of media reports covering human brain organoid biotechnology. We hypothesize that media coverage of brain organoid research is dominated with utopian and dystopian scenarios which fit more with science fiction than with science fact.

## Study design and methods

Our study sought to investigate how human brain organoid research is presented in media literature, with particular focus on how potential uses and ethical issues are discussed. To do so, we designed a structured review and scoring schema to qualitatively analyze the treatment of brain organoids in media literature. We conducted our search on November 26, 2019 using the NexisUni database, and relevant sources were collected using the following search terms: ((brain OR cerebral) AND (organoid)) AND (ethic* OR moral* OR social). We then filtered the search to include sources we loosely defined as media for general public consumption. Namely, we sought to exclude peer-reviewed articles and empirical studies targeted towards experts in the field and/or those with extensive foundational knowledge regarding biotechnological advancements such as these. Instead, we were looking for any literature generally accessible, digestible, and targeted towards those without such backgrounds. Of the available filters, we chose to include “Newswires and press releases”, “newspapers”, “news transcripts”, “magazines and journals”, “weblinks”, “Industry trade press”, “web-based publications”, “undefined” and “aggregate news sources”. We then applied exclusion criteria of tangentiality, or articles mentioning organoids only in passing, (*n* = 30, 30%) and duplication (*n* = 69, 70%) to cut out 99 texts, leaving us with a sample 92 sources. On August 21, 2020 we repeated the search to span November 27, 2019 - December 31, 2019 and updated the dataset. This added four sources (sources 127–130), one of which was a duplicate and two of which were excluded as tangential to the topic. This increased the sample size to 93 sources. From this, we created a pilot sample (10% of the size of our sample) coded by two independent coders (A.P. and a research assistant under supervision from the corresponding author) to establish intercoder reliability. Any discrepancies in coding of the pilot sample was discussed and consensually resolved with the help of a third party (V.D.).

**Fig. 1:**
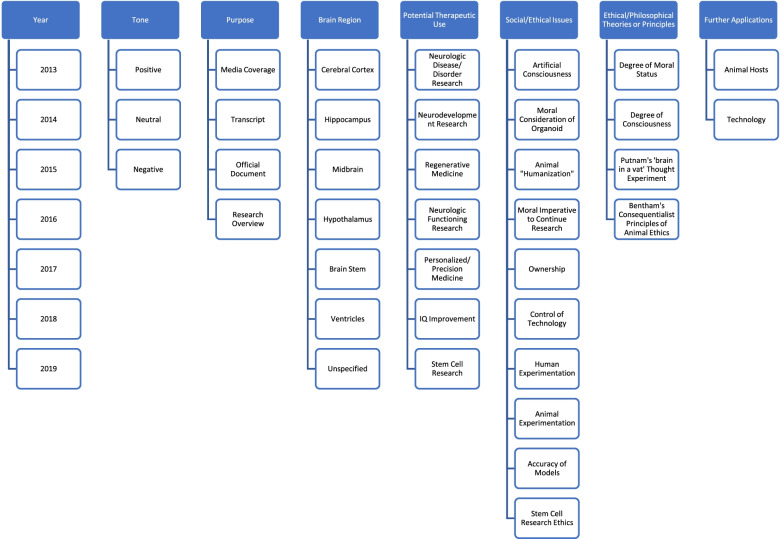
Codes and Subnodes used for Media Sample. Flow chart of eight codes and corresponding subcodes used to code pilot sample and overall sample

Both presumptive deliberation and this pilot sample helped inform our coding scheme of the following eight categories for each article: 1) Year published, 2) Tone of Article, 3) Purpose of Text, 4) Brain region targeted by the organoid, 5) Potential therapeutic use, 6) Social/Ethical issues, 7) Ethical/Philosophical theories or principles mentioned, 8) Further applications. Subcategories were associated with some criteria. For full description and code examples, see Fig. 1. A list of sources is included in Supplemental Materials.

***Year*** referred to the when the text was published. The ***tone*** category consisted of “positive”, “neutral”, or “negative” articles. Positive articles focused primarily on the potential benefits of organoid research, while disregarding or briefly discussing associated ethical concerns. Neutral articles addressed both therapeutic potential and the ethical context surrounding the research, and negative articles focused on social and ethical questions raised by brain organoid research and applications. ***Purpose*** was used to categorize the type of text and intended audiences. ***Brain region*** categorization was used to highlight target brain regions mentioned, if specified, to designate clinically significant relationships between structure and function, which could be relevant for particular disease and drug pathways. Of note, the “cerebral cortex” is responsible for higher level functioning such as planning, emotional regulation, and problem solving; the “hippocampus” is responsible for memory formation and storage; and the “hypothalamus” regulates motivational states (i.e. feeding, fighting, etc.).

The ***potential therapeutic use*** subcategory informed us of what clinical applications were being discussed. “Neurodevelopment research” was used to classify articles that explained how brain organoid might be used to better understand developmental processes, particularly those of the brain. Meanwhile, articles discussing the potential of brain organoids to illuminate and model various disease or disorder processes and treatment pathways were coded as “neurologic disease/disorder research”. Articles that detailed how the organoids may be used to better understand neural connections and pathways within the brain were coded as “neurologic functioning research”. Articles mentioning “personalized or precision medicine” discussed how the organoids may be used to develop individualized drug treatment plans. Those coded “regenerative medicine” outlined the potential of organoids to allow regeneration of patients’ cells, tissues, and even organs for repair or transplantation. Some articles mentioned the potential for “IQ improvement”, while some articles mentioned “stem cell research” as a potential organoid application.

Discussion of ***social or ethical issues*** was evaluated using “moral considerations of the organoid”, which referred to articles musing on whether the brain organoids or chimeras should be given unique moral and/or legal consideration given their potential for sensation and consciousness. The related subcategory of “artificial consciousness” coded for the idea that organoids/chimeras might develop independent consciousness. “Animal “humanization’” referred to concerns that the chimeras might develop human-like capacities following the implantation. Issues of “ownership” called for guidelines surrounding consent and distribution. Articles discussing “control of technology” speculated uncontrolled growth of the organoid, causing potential complications. “Accuracy of models” referred to the validity of the organoids for use in clinical and therapeutic contexts. “Stem cell research ethics” coded for texts that discussed ethical concerns of tissue procurement. “Animal experimentation” expressed concerns with chimera use and discussed the current state of animal models, while “human experimentation” discussions were centered on the current use of human research subjects. Articles calling for a “Moral imperative” to continue research cited the need to develop organoid research to alleviate suffering related to neurologic conditions.

The ***ethical/philosophical theories or principles***, if mentioned, included “degrees of moral status” and “degrees of consciousness”, the former referring to the criteria used to designate ethical considerations among organisms, and the latter to how varying levels of consciousness are defined and regarded. The subcategory “Hilary Putnam’s ‘brain in a vat’ thought experiment” referred to discussion of whether a brain placed in life-sustaining fluids and connected to a sensation-perceiving supercomputer might be considered human. Finally, “Bentham’s consequentialist theory of animal ethics” outlined guidelines for ethical considerations of animals based on suffering.

Finally, the ***further applications for organoid*** category referred to either “animal host applications”, or chimeras, or “technology implications”, referring to “organ on a chip”, artificial intelligence, and technological advances related to brain organoid research.

## Results

### Increase in publication by year

**Fig. 2:**
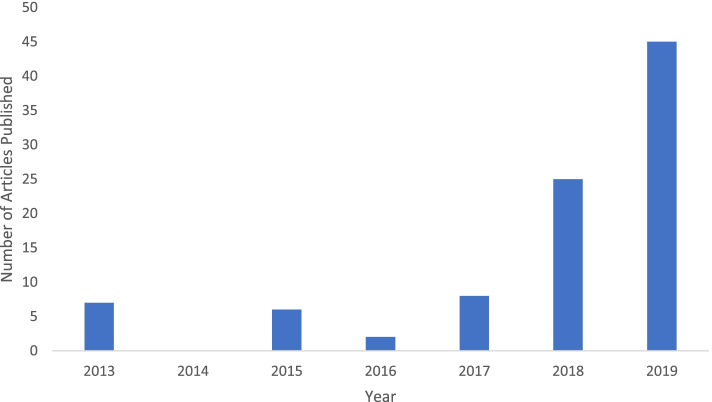
Upward Trend in Media Samples Over Time. Histogram of media sample publications by year. *n* = 7, 0, 6, and 2 for 8%, 0%, 6%, and 2% of the sample in years 2013–2016, respectively. *n* = 8, 25, and 45 for 9%, 27%, and 48% of the sample in years 2017–2019, respectively

From 2013–2016 there were only 15 media reports on brain organoids (Fig. 2). There was a notable increase in media sources in 2019 (*n* = 45, 48%), as publications discussing brain organoid coverage in media literature shared a visible upward trend with years between 2016 and 2019 (Fig. 2). The second most common publication year was 2018 (*n* = 25, 27%), followed by 2017 (*n* = 8, 9%), and 2016 (*n* = 2, 2%). Interestingly, there was an increase in frequency of articles in 2015 (*n* = 6, 6%) and 2013 (*n* = 7, 8%), yet our sample contained no articles published in 2014 (*n* = 0, 0%). Considering the high volume of articles in 2019 as compared to 2013–2018, our analysis of tone will focus primarily on comparing these two time periods. It was surprising that our sample contained no sources from 2014, especially considering that multiple articles were published in 2013 (*n* = 7) and 2015 (*n* = 6). One possible explanation could include that the technology was too recent at this time to have much relevance to the general public.

### Prevalence of media coverage texts

Unsurprisingly, the vast majority of texts were media coverage (*n* = 85, 91%). Of the remaining texts, transcripts were most common (*n* = 5, 5%), followed by official documents (*n* = 2, 2%) and research overviews (*n* = 1, 1%)

### Brain region

Most articles (*n* = 62, 67%) did not specify the brain region associated with the brain organoid. Of the sources that did specify a brain region (*n* = 33, 35%), the most commonly discussed was the cerebral cortex (*n* = 29/31, 94%), followed by the hippocampus (*n* = 4/31, 13%) and the hypothalamus (*n* = 3/31, 10%). The brain stem, ventricles, and midbrain (*n* = 1/31, 3% each) were sparsely mentioned. All three are forebrain regions, suggesting that the forebrain is either technically simpler to mimic with an organoid or is of particular interest. However, it is also important to note the complexity of many of these structures, especially within the context of the entire neural network, and the associated difficulty of successfully recapitulating these regions as organoids.

### Tone

**Fig. 3:**
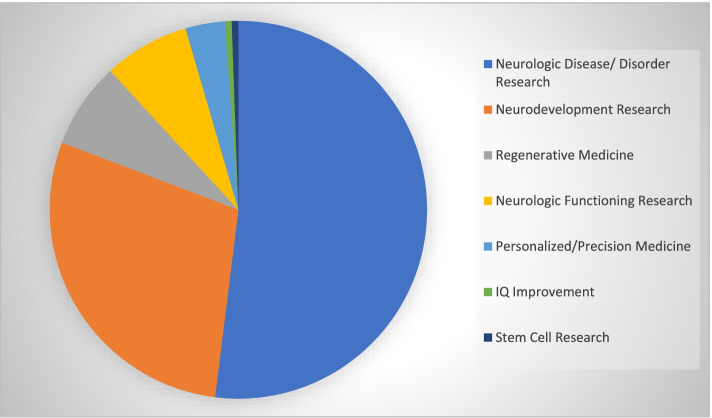
Increased Frequency of Polarized Tone of Media Sample with Year. Histogram of media samples each year based on tone. For positive, neutral, and negative respectively, *n* = 4, 3, and 0 in 2013, *n* = 0 for each category in 2014, *n* = 5, 1, 0 in 2015, and *n* = 2, 0, 0 in 2016. Likewise, for positive, neutral, and negative articles respectively, *n* = 1, 5, and 2 in 2017, *n* = 7, 18, and 0 in 2018, and *n* = 11, 30 and 4 in 2019

Article tone was categorized as positive, negative or neutral. The majority of the articles were neutral (*n* = 57, 61%). However, positive articles (*n* = 30, 32%) were far more frequent than negative articles (*n* = 6, 6%). From 2013–2016 positively toned articles were relatively infrequent, especially as compared to neutral texts (Fig. 3). However, beginning in 2017 and rapidly increasing through 2019, positively toned articles increased in frequency. Meanwhile, negatively toned articles also increased in frequency from 2017 to 2019, though less dramatically than positive articles. In fact, negative articles did not appear in media literature until 2017.

### Focus on neurologic disease and neurodevelopment research as potential therapeutic uses

**Fig. 4:**
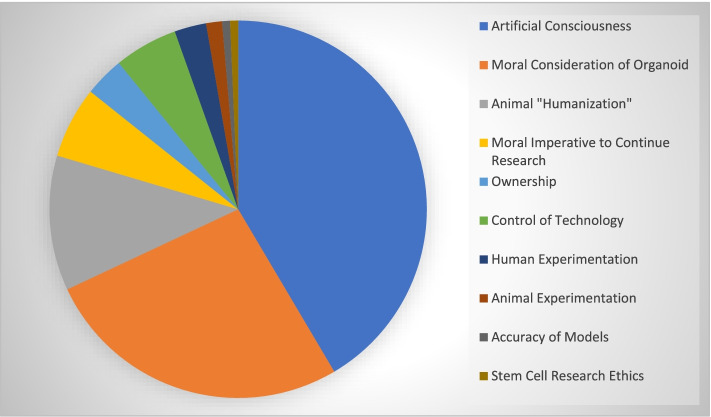
Percentages of Therapeutic Uses as Discussed in Media Sample. Pie chart of percent frequencies of different therapeutic uses in media sample. Of note, many samples discussed multiple potential therapeutic uses, thus percentages are comparative to one another, rather than out of the sample as a whole. *n* = 86, 47, and 12 for 92%, 51%, and 13% of samples discussed neurologic disease/disorder research, neurodevelopment research, and regenerative medicine respectively. *n* = 12, 6, 1, and 1 for 13%, 6%, 1% and 1% of samples discussed neurologic functioning research, personalized/precision medicine, IQ improvement and stem cell research respectively

Many articles discussed neurologic disease/disorder research as a potential therapeutic use (Fig. 4) (*n* = 86, 92%). Neurodevelopment research was also a common topic (*n* = 47, 51%). Regenerative medicine was another frequently discussed application (*n* = 12, 13%), as was neurologic functioning research (*n* = 12, 13%), which were followed by personalized/precision medicine (*n* = 6, 6%), IQ improvement and stem cell research (*n* = 1, 1% each). Table 2 gives example quotations from various texts in our sample to help illustrate how the potential therapeutic use subcategories are discussed in the sources.

**Table 2:** Example Quotes of Potential Therapeutic Uses from Media Sample

Potential Therapeutic Uses	Quotation from Media Sample Texts
Neurodevelopmental Research	"What these cerebral organoids excel at, he says, is offering a picture of how the brain develops, and how that development can go wrong" (source 95).
Neurologic Disease/Disorder Research	"But [the minibrains] also promise hope of a cure for illnesses ranging from childhood epilepsy to *Alzheimer's disease* and brain cancer" (source 28).
Neurologic Functioning Research	"there is the broader intellectual quest to understand mysteries such as memory, emotion and consciousness by studying synthetic brains” (source 62).
Personalized/Precision Medicine	“Organoids will bring precision medicine closer to reality by developing patient-specific treatment strategies by studying which drugs the patient is most sensitive to" (source 31).
Regenerative Medicine	"Leaving the controversial and ethical issues aside, the fact that you might be able to grow your own organ that has your own genes in a lab and transplant it when needed, avoiding the search for a donor and the immune reaction that happens after the transplant, is overwhelming" (source 45).
IQ Improvement	"The scientist claimed that using this technique to bolster brain matter and improve someone's IQ would be 'quite safe'"(source 40).
Stem Cell Research	"organoids are expected to advance our understanding of tissue renewal, stem cell/niche functions and tissue responses to drugs, mutation or damage, as well as unlocking the mysteries of several brain diseases and neurological disorders” (source 85).

### Social/Ethical focus on artificial consciousness and moral considerations

**Fig. 5:**
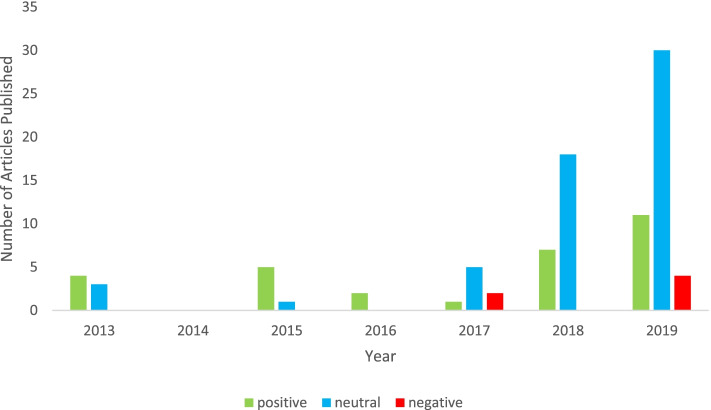
Percentages of Social/Ethical Issues as Discussed in Media Literature. Pie chart of percent frequencies of different social/ethical issues discussed in media sample. Of note, many samples discussed multiple issues, thus percentages are comparative to one another, rather than out of the sample as a whole. *n* = 57, 36, 16, and 8 for 61%, 39%, 17% and 9% of samples discussed artificial consciousness, moral considerations of the organoid, animal “humanization”, and moral imperative to continue brain organoid research. *n* = 5, 7, 4, 2, 1, and 1 for 5%, 8%, 4%, 2%, 1%, and 1% of samples discussed ownership, control of technology, human experimentation, animal experimentation, accuracy of models, and stem cell research respectively

As shown in Fig. 5, artificial consciousness was the most common ethical issue discussed among our sample (*n* = 57, 61%). Many articles also discussed moral considerations of the organoid (*n* = 36, 39%). Of slightly lesser frequency were issues of animal “humanization” (*n* = 16, 17%) and moral imperatives to continue brain organoid research (*n* = 8, 9%). Ownership (*n* = 5, 5%) and control of technology (*n* = 7, 8%) were both discussed relatively infrequently, as was human experimentation (*n* = 4, 4%). Finally, animal experimentation (*n* = 2, 2%), accuracy of models (*n* = 1, 1%), and stem cell research (*n* = 1, 1%) were least frequently discussed in our sample. Table 3 gives example quotations from the media sample to illustrate how the social and ethical issues are discussed in the sources.

**Table 3:** Example Quotes of Social/Ethical Issues Discussed in the Media Sample

Social/Ethical Issues	Quotations from Media Sample Texts
Moral Consideration of Organoid	"Through this line of thinking, if minibrains developed sentient capacities similar to "real" brains, they could, in essence, hold the same consciousness of humans and thus potentially have human rights" (source 61).
Artificial Consciousness	"Is it possible that an organoid far off in the future could develop something that looks like consciousness or any kind of sentience, the ability to feel something like pain or experience anything" (source 116).
Animal “Humanization”	"once the door is open, you can come to all sorts of scenarios including the 100 per cent humanisation of an animal's brain, and all the ethical concerns that raises" (source 37).
Ownership	“'If I take a snippet of cells from your arm, make stem cells, and make an organoid in a dish, do you still own it? Does my lab? My university?'" (source 124).
Control of Technology	“It might be that the technology is not ready yet, or we don't know how to control the technology" (source 126).
Accuracy of Models	“Another central issue is... how true to life an in vitro model of human development needs to be in order to be both scientifically valuable and ethically acceptable" (source 85).
Stem Cell Research	"There are concerns that, as lab-grown cultures become increasingly indistinguishable from a human brain, researchers could violate ethical codes of conduct around stem cell experimentation" (source 53).
Animal Experimentation	“More sophisticated in-vitro models could replace the need to have animal models or human foetal tissue in future research" (source 53).
Human Experimentation	“The line between research on organoids and human experimentation, however, is unclear and remains to be established" (source 70).
Suffering from Neurologic Disease/Disorders	"there is a mandate to keep pushing, not least because of what it might mean to the world at large: more diseases combated, more treatments developed, more lives saved and, above all, a fuller glimpse of a dauntingly complex organ" (source 94).

### Ethical/Philosophical theories or principles mentioned

Many articles (*n* = 81, 87%) did not mention an ethical or philosophical theory or principle. Among those that did, degrees of moral status was the most common (*n* = 8, 9%). This subcategory was followed by Putnam’s “brain in a vat” thought experiment (*n* = 4, 4%), degrees of consciousness (*n* = 3, 3%), and finally Bentham’s consequentialist theory of animal ethics (*n* = 1, 1%).

### Applying organoids to animal hosts and Implications for technology

Discussion of creating animal chimeras using brain organoid technology was discussed more frequently (*n* = 32, 34%) than technological applications (*n* = 9, 10%). This places animal chimera applications at the forefront of both the ethical and developmental media treatment of brain organoids.

## Discussion

In this study, we set out to better understand how the general public is being informed about brain organoids by assessing how the technology is portrayed in media literature. Our investigation sought to understand what promises are presented surrounding brain organoids research, how these promises are contextualized by current brain organoid research, and where this conversation is situated amongst relevant ethical and social issues. The small (*n* = 93) final sample size was to be expected considering the novelty of brain organoid technology and in some ways limits interpretation. Yet, the history and trajectory of this media portrayal can help predict and understand future communication in this dynamic field of science and technology. The brain organoid technology itself was first introduced within the last few years and, though it is rapidly developing, is just being established within media literature.

### Trends in media reports

Regarding article type, considering our study was focused on media coverage, hoping to evaluate how information regarding brain organoids is being presented through media literature, it is reasonable that our sample contained a vast majority of texts that were labeled ‘media coverage’ (91%). There were few ‘official documents’ in our sample (2%), implying that the topic was not being heavily discussed among government and policy-makers within the date range of which our search was conducted. This could have implications for regulation of the technology, or lack thereof, which could in turn work to spark public concern and enhance fears. It could also be a result of the search criteria of our study, which was biased towards generalized, less targeted media literature. Alternatively, these results could be a reflection of the relative novelty of the technology and its applications. Regardless, moving forward it is important that the complexity of the research is understood and discussed amongst not only the scientific community and public, but also those in positions of power (concerning funding, regulation, etc.).

Our analysis found that the number of media publications on brain organoid technology increased from initial discovery to the end of the search range (2013–2019) with nearly half (*n* = 45, 48%) of our sample being published in 2019 alone. This indicates that the technology is rapidly developing and attracting scientific attention since its introduction. It is thus uncontroversial to posit that public-directed media sources have increasingly gained interest in the topic within the last few years as brain organoids become more advanced and potentially relevant in clinical and scientific settings. When it was first introduced in 2013, it likely gained public attention for the novelty and potential offered. However, with problems such as vascularization, described above, there were many challenges left to be resolved before brain organoids could truly be imagined in a public arena. As the technology was further refined and developed in the following years, it once again generated media excitement.

### Polarization

Increasing media representation of novel neuroscientific techniques can be both beneficial and detrimental. On the one hand, it can increase appreciation of novel scientific findings and pave the way for implementation of therapies. However, if the media representation is skewed or polarized, it can lead to a public backlash and starve the public funding for research at a crucial time in this nascent field of science. Our findings suggest that there is a rising trend in neutral media coverage of human brain organoids, and that positive coverage outweighs negative coverage. However, the sharp discrepancy between negative and positive reports points to the lurking danger of polarization. Recent headlines like “Bioengineered hippocampal organoids for epilepsy treatment project” (source 11) and “Growing new body parts can have enormous benefits…” (source 76) imply exciting benefits of brain organoids, many of which are speculative and could lead an uninformed public to expect cures or new abilities. On the other hand, headlines like “Rats with HUMAN brains- human/rodent hybrids created in lab spark ethical nightmare” (source 83) might scare the public with dystopian images that are not a current scientific reality. Discussions centered around such topics are prone to evoking feelings of deep-seated discomfort and disgust among readers. Some have suggested such distaste is common surrounding biotechnologies that seem to violate biological laws governing the natural world (Niemelä, (Niemelä 2011)). Brain organoids would certainly fit within this cognitive category, especially when moving into discussions of chimerism. Thus, one would expect initial discomfort with the technology due to its significant novelty. This then calls for education on the topic and its current capacities, and again necessitates accurate, fact-based dissemination of information. Sources with strong negative language that describe hybrid scenarios unfounded in scientific fact work to evoke fear and disgust while avoiding useful information regarding what brain organoids are, how they are being used, and their current limitations. Thus, overall, our results point to a picture of a relatively immature (i.e., prone to hyping or catastrophizing) media discussion of brain organoids, which to a certain extent suggests a lack of sufficient applied ethics scholarship on the topic.

### Overpromising

While the existing research is promising, it is still in the beginning stages of application and evaluation. Considering how recent brain organoid research and development is, it is difficult to draw well-supported, firm conclusions from the data as it currently stands, especially as it relates to clinical settings. As such, it is important to accurately portray scientific results to the public and avoid pitfalls like overgeneralization of results and inferring uses for the organoids not fully supported by the science. Pitfalls like these could lead the public to expect a ‘cure all’ technology that is not a reality, and less feasible speculations about the research such as transplantable brain regions or cures for diseases like cancer and dementia, giving false hope to already struggling patients and families. The resulting disillusion could then pose an obstacle to further public support for the research. This calls for a balanced approach to the topic in media literature that takes into account both scientifically grounded ethical issues *and* potential therapeutic uses, such that the public would have an informed understanding of brain organoid research, and the biotechnology is able to ethically advance to its fullest potential.

Our results indicated that ‘neurologic disease research’ (92%) and ‘neurodevelopment research’ (51%) were the most commonly discussed potential therapeutic uses. This is reassuring, considering brain organoids have already been successfully applied and are actively being used in these research areas (Koo et al., (Koo et al. 2019)). As such, media sources discussing these therapeutic applications for organoids are well-supported by scientific research. However, some sources also discussed topics such as ‘regenerative medicine’ (13%) and ‘IQ improvement’ (1%), which are widely speculative and not a current scientific reality for brain organoids. It is important that when discussing such applications, literature emphasizes the fact that they are *possibilities* to be explored in the future, but are not founded by organoid technology as it currently stands. This avoids giving false promise to readers and “overhyping” the technology before it has been fully developed.

An additional caution to observe when discussing potential therapeutic uses of brain organoids is how language is used surrounding these applications, which can be particularly influential. This especially relates to discussions of ‘neurologic disease/disorder research’ and ‘neurodevelopmental research’, both of which are inexplicably tied to significant neuropathologies. Namely, it is important that the research is presented as offering an improved understanding of disease and drug pathways and development, rather than promising a cure to related diseases and disorders. While this research does contribute to development of treatment, the technology does not currently reflect the ability to fully cure neurologic disease and disorders.

### Sensationalized

Despite the fact that the development of consciousness in brain organoids is technically impossible at this stage in technology and unrealistic in the foreseeable future, the issue dominating the media discussion is ‘artificial consciousness’ (61%). It is somewhat reassuring that the ‘moral consideration of organoids,’ an issue that is broadly correct but of unclear level of relevance at this stage, is second (with 39%). ‘Animal “humanization”’, similar to ‘artificial consciousness’ is also fairly commonly discussed in media literature (17%), yet relatively unfounded in current research. While many sources took an overall neutral tone, most expressed some level of concern over these issues, noting that while the science may not support development of consciousness or humanization now, it would be reasonable to expect these issues to arise in the future as the technology advances. However, many also fail to mention the complexity and abstraction inherent in measuring ideas such as consciousness and moral status. This could work to inspire ungrounded fear in the public, as media sources point towards fully conscious organoids or part human animals as a near possibility or inevitability. Ethical concerns of moral status and regulation are relevant, especially as brain organoid technology further develops, but must be addressed in a manner consistent with scientific reality.

Meanwhile, concerns of ‘animal experimentation’ (2%), ‘ownership’ (5%), ‘control of technology’ (8%), ‘stem cell research ethics’ (1%) and ‘accuracy of models’ (1%) were all discussed much less frequently within the media literature. Such discrepancies between topics such as these and those concerning consciousness and ‘animal “humanization”’ imply that media portrayal of relevant ethical issues associated with brain organoids is misplaced. Emphasis is placed on current scientific impossibilities, rather than calling for regulatory guidelines and ethical practice within the research moving forward (i.e. procurement of materials, informed consent, validity and replicability), which are far more relevant given the current state of research. Media literature should thus work to address more scientifically grounded concerns of regulation of brain organoid technology, such that it may advance in a safe and ethically relevant way.

The vast majority of articles did not address relevant ethical or philosophical theories or principles in their discussion of brain organoid technology. This again suggests that media representation of the technology lacks ethical and/or philosophical support in addition to scientific grounding. The most common philosophical principle mentioned was ‘degrees of moral status’ (9%), which tied into the discussion of ‘moral status of organoids’. This ethical issue, while valid, may be less relevant than other issues less commonly discussed.

Taken together, our findings warn of increasing polarization. Many sources describe promises and ethical concerns unfounded in current research, exhibiting misplaced concern. Given the inhibitory effects public fear or disillusion can have on the progress of research coupled with efforts to prevent a similar development as that of stem cell research in the U.S., it is important that media literature provides an accurate reflection of therapeutic potential and ethical issues regarding brain organoids. We suggest that follow-up reviews seeking to understand how both the research and portrayal of brain organoids have changed would be both useful and politically relevant in the next 5–10 years or sooner. Additionally, studies focusing on portrayal of brain organoids within government and political contexts could be useful, to help evaluate how the research is being portrayed and discussed at policy-making and funding levels.

## Conclusion

A typical approach in applied ethics for any kind of new science and technology is to discuss concerns of various levels of urgency. As brain organoid technologies continue to develop and applications widen, media literature will continue to follow suite with increasing coverage and interest, as evidenced by our upward trend in sources with time. Our results suggest that while *most* topics being addressed are relevant to some degree, there seems to be a general media preoccupation with less feasible applications and ethical concerns. For example, while potential organoid consciousness is of interest, it is misplaced as the dominant ethical issue within media literature, considering technology limitations as they stand. Issues of stem cell donor consent and regulatory guidelines are much more relevant to the current state of the technology (Yeager, (Yeager 2018)), but are much less frequently discussed. Similarly, the possibility of animal humanization is reasonable and concerning, but is again subject to brain organoid limitations, which include a lack of higher-order functional capacities and layered organization (Chiaradia and Lancaster, (Chiaradia and Lancaster 2020)). As Chen and colleagues ((Chen et al. 2019)) argue, the potential for “specific brain enhancements” and related changes to moral status are more relevant and pressing than assumptions of full animal humanization. Our contribution to this debate is to parse out real and urgent issues (e.g., the increase in invasive animal experimentation, ownership and control over brain organoid technology as well as issues with accuracy of models), from overblown fears and non-issues. Media presentation of brain organoid research tends towards shallow and sensationalistic representations, citing issues that are of little relevance and ethical concern at this stage in the research and thus pushing readers towards misinformed discomfort and fear. Simultaneously, more relevant ethical and regulatory concerns are being discussed at much lower volumes, if at all. Going forward, we suggest that media literature should work to take less biased and more fact-based approaches to brain organoid information dissemination, such that maximal clinical benefit may be explored while addressing relevant ethical, social, and philosophical concerns.

## Supplementary Information

**Additional file 1. **List of Media Sources.

## Data Availability

The datasets analyzed during the current study is available from the corresponding author on reasonable request.

## Abbreviations

3D: three dimensional
PCR: polymerase chain reaction
IQ: Intelligence quotient
