# Supplementary material for: Media portrayal of ethical and social issues in brain organoid research
Source: Philos Ethics Humanit Med. 2022 Apr 13;17:8. doi: 10.1186/s13010-022-00119-z (PMC9006586; doi:10.1186/s13010-022-00119-z)
Supplement: Supplementary file 1 — Additional file 1. List of Media Sources. [file 13010_2022_119_MOESM1_ESM.docx]

(Supplemental Materials)

**List of Media Sources**

1. $8.5 Million Gift Supports University of California-Santa Cruz Genomics Institute, Targeted News Service, Targeted News Service, (October 13, 2019 Sunday)
2. A HUMAN BRAIN MODEL IN A PETRI DISH?, States News Service, States News Service, (January 15, 2019 Tuesday)
3. Jon Hamilton. (August 29, 2019 Thursday). After Months In A Dish, Lab-Grown Minibrains Start Making 'Brain Waves'. NPR Shots. Retrieved from https://advance-lexis-com.prox.lib.ncsu.edu/api/document?collection=news&id=urn:contentItem:5WXP-08F1-F105-G1SH-00000-00&context=1516831.
4. After Months In A Dish, Lab-Grown Minibrains Start Making 'Brain Waves', NPR All Things Considered, (August 29, 2019 Thursday)
5. Targeted News Service. (December 4, 2017 Monday). Alcoholism: Clinical & Experimental Research Issues 19 Research Articles in December 2017 Edition. Targeted News Service. Retrieved from https://advance-lexis-com.prox.lib.ncsu.edu/api/document?collection=news&id=urn:contentItem:5R3P-KXF1-DYG2-R3TY-00000-00&context=1516831.
6. A M I N I I N REVOLUTION BRAIN SCIENCE; Grown in laboratories from stem cells, 'mini-brains' are helping us to understand how human brains develop, age and respond to treatment. Clive Cookson meets the neuroscientist who pioneered the work, Clive Cookson, Financial Times (London, England), FT WEEKEND MAGAZINE;FEATURES; Pg. 30,31,32,33,35, (September 7, 2019 Saturday)
7. A patient's budding cortex -- in a dish?, National Institutes of Health Documents and Publications, NATIONAL INSTITUTES OF HEALTH - NIH, (May 28, 2015)
8. August 28, 2019: North Carolina Biotechnology Center: Pyxus Inks Deal with Cornell U to Study Hemp Cultivation, News Bites- Private Companies, ANNOUNCEMENTS, (August 29, 2019 Thursday)
9. ‘Baby’ brains made in the lab: Even scientists behind the breakthrough are astonished, WebNews- English, (August 31, 2019 Saturday)
10. BIG IDEAS IN HEALTH AND SCIENCE FOR 2016, State News Service, (December 11, 2015 Friday)
11. (December 11, 2018 Tuesday). Bioengineered hippocampal organoids for epilepsy treatment Project. Pivotal Sources. Retrieved from https://advance-lexis-com.prox.lib.ncsu.edu/api/document?collection=news&id=urn:contentItem:5TY0-FHK1-JDKC-R311-00000-00&context=1516831.
12. Blob-like brains created in lab could have 'thoughts' and are 'suffering', scientists warn; MINIATURE brains which have been grown in the lab could have some form of consciousness and could be suffering as a result, scientists have worryingly claimed., Sean Martin, Express Online, (October 22, 2019 Tuesday)
13. Brain activity like that of humans found in lab-grown 'mini brains' study, WebNews- English (August 31, 2019 Friday)
14. Brain Changes in Autism: Specific Cell Types, FARS News Agency, (May 20, 2019 Monday)
15. Brain teaser; LEADING ARTICLE 'Cerebral organoids' have much to teach us, The Independent (London), COMMENT; Pg. 16, (August 29, 2013 Thursday)
16. Brainstorming the ethics of neuroscience research in the age of organoids, Iran Daily, (April 29, 2018 Sunday)
17. Brain waves detected in lab grown mini-brains, AFP, Daily Nation (Kenya), (August 29, 2019 Thursday)
18. Briefing.com: Hourly In Play (R) - 17:00 ET, Briefing.com, Comtex News Network, (March 27, 2019 Wednesday)
19. Building a Better Brain-in-a-Dish, FARS News Agency, (September 9, 2018 Sunday)
20. Can organoids, derived from stem cells, be used in disease treatments?, WebNews- English, (October 26, 2019 Saturday)
21. 'Cerebral organoids' have much to teach us; For all our intelligence, we are still far from sure how the brain works, Editorial, Independent.co.uk, VOICES, (August 28, 2013 Wednesday)
22. CHIMPANZEE 'MINI-BRAINS' HINT AT SECRETS OF HUMAN EVOLUTION, States News Service, States News Service, (February 7, 2019 Thursday)
23. Targeted News Service. (July 31, 2019 Wednesday). Clark University: Ibrahim Ozgenc's Cancer Research is More Than Targeted -- It's Precise. Targeted News Service. Retrieved from https://advance-lexis-com.prox.lib.ncsu.edu/api/document?collection=news&id=urn:contentItem:5WRH-CCV1-JC11-1228-00000-00&context=1516831.
24. Cloning monkeys for research puts humans on a slippery ethical slope, David Hunter, Associate Professor of Medical Ethics, Flinders University, The Conversation - Australia, (February 1, 2019 Friday)
25. Cold Spring Harbor Laboratory: Of Mice and Model Organisms, Targeted News Service, Targeted News Service, (July 31, 2019 Wednesday)
26. Company plan to resurrect the dead with stem cells, WebNews- English, (October 21, 2019 Monday)
27. Targeted News Service. (April 11, 2018 Wednesday). Corning to Highlight Cancer Research Innovations Driven by 3D Cell Culture at AACR 2018. Targeted News Service. Retrieved from https://advance-lexis-com.prox.lib.ncsu.edu/api/document?collection=news&id=urn:contentItem:5S59-3HR1-DYG2-R3VS-00000-00&context=1516831.
28. Could lab-grown human minibrains help treat Alzheimer's and epilepsy?, Paul Biegler, The Sydney Morning Herald (Australia) - Online, (November 19, 2018 Monday)
29. (July 8, 2019 Monday). Cultivators Seek to Maximize the Cannabis Plant's Potential; FinancialBuzz.com News Commentary. PR Newswire. Retrieved from https://advance-lexis-com.prox.lib.ncsu.edu/api/document?collection=news&id=urn:contentItem:5WHP-VD01-DXP3-R0HF-00000-00&context=1516831.
30. CUMC Celebrates 2015-2016, Issue 5, US Official News, (May 18, 2016 Wednesday)
31. Daily Health News Bulletin (28-10-19)., WebNews- English, (October 28, 2019 Monday)
32. Disembodied Brains Are Scary Or How Sci-Fi Influences Science, WebNews- Academic, (November 23, 2019 Saturday)
33. (January 25, 2019 Friday). -Eindhoven University of Technology: TU/e leads project for developing nervous system-on-a-chip. ENP Newswire. Retrieved from https://advance-lexis-com.prox.lib.ncsu.edu/api/document?collection=news&id=urn:contentItem:5V8J-WRR1-F0K1-N037-00000-00&context=1516831.
34. Ethical posers, Gulf Times, (April 29, 2018 Sunday)
35. Ethics debate overdue in human brain research: experts, Agence France Presse -- English, (April 25, 2018 Wednesday)
36. Explainer: Why scientists are growing 'mini human brains', and why this might be unethical, gordon.feeney, Today (Singapore) - Online, Singapore; Singapore, (October 21, 2019 Monday)
37. Frankenrats: human brain cells implanted into rodents, Oliver Moody, The Times (London), NEWS; Pg. 11, (November 9, 2017 Thursday)
38. Frankenstein cyborg CRABS? Artificial intelligence researchers are putting Neanderthal brains into ROBOTS; A US scientific study is trying to find out why Neanderthals went extinct, By Jeff Parsons, irishmirror.ie, SCIENCE; Version:1, (June 28, 2018 Thursday)
39. Fresh urgency in mapping out ethics of brain organoid research, Julian Koplin, Research Fellow in Biomedical Ethics, Melbourne Law School and Murdoch Children's Research Institute, University of Melbourne, The Conversation - Australia, (November 21, 2018 Wednesday)
40. From farming pigs for organs to human brain transplants: Controversial geneticist George Church predicts the future of mankind, HARRY PETTIT FOR MAILONLINE, MailOnline, SCIENCE; Version:1, (July 3, 2018 Tuesday)
41. Growing brains in labs: why it's time for an ethical debate; Experts argue that experiments have edged so much closer to the possibility of consciousness that guidelines are needed, Ian Sample Science editor, The Guardian(London), SCIENCE; Version:1, (April 25, 2018 Wednesday)
42. House Appropriations Committee Issues Report on Departments of Labor, HHS, Education, Related Agencies Appropriations Bill, 2018 (Part 4 of 10), Targeted News Service, Targeted News Service, (July 27, 2017 Thursday)
43. House Government Reform Subcommittees Issues Testimony From Charlotte Lozier Institute, Targeted News Service, Targeted News Service, (December 14, 2018 Friday)
44. (December 13, 2018). House Oversight and Government Reform Subcommittee on Government Operations and Health Care, Benefits and Administrative Rules Hearing; "Exploring Alternatives to Fetal Tissue Research."; Testimony by Tara Lee, Associate Scholar, Charlotte Lozier Institute; WAXc11c1318H014. Congressional Documents and Publications. Retrieved from https://advance-lexis-com.prox.lib.ncsu.edu/api/document?collection=news&id=urn:contentItem:5TYM-H7R1-DYVR-P2H9-00000-00&context=1516831.
45. Human brain grown in lab to study diseases, Gulf Weekly, (August 26, 2015)
46. Human-like neural activity detected in lab-grown mini brains, Iran Daily, (August 30, 2019 Friday)
47. Human mini-brains made from stem cells make brain waves like preemies, WebNews- Academic, (August 30, 2019 Friday)
48. In creating so-called mini-brains, how close to a real human brain is too close?, Global English (Middle East and North Africa Financial Network), (October 13, 2019 Sunday)
49. Targeted News Service. (July 25, 2019 Thursday). Initiative Co-Led By University College London to Unlock Secrets of Human Development. Targeted News Service. Retrieved from https://advance-lexis-com.prox.lib.ncsu.edu/api/document?collection=news&id=urn:contentItem:5WNC-VBD1-JC11-143J-00000-00&context=1516831.
50. Journal of Visualized Experiments Issues 114 Research Articles in September 2019 Edition, Targeted News Service, Targeted News Service, (October 2, 2019 Wednesday)
51. Lab-grown Mini Brains Produce Human-like Brain Waves, WebNews- English, (August 30, 2019 Friday)
52. Lab-grown brains start producing human-like 'brain waves', By Tom Hoggins, telegraph.co.uk, TECHNOLOGY INTELLIGENCE; Version:1, (August 30, 2019 Friday)
53. Lab-grown mini-brains produce signals just like those in pre-term babies, Jacklin Kwan, The Straits Times (Singapore), SCIENCE, (August 31, 2019 Saturday)
54. Lab-grown mini brains: we can't dismiss the possibility that they could one day outsmart us, Impact News Service, (November 5, 2019 Tuesday)
55. Lab-grown human brains spark fears of 'evil consequences', WebNews- English, (August 29, 2019 Thursday)
56. MIL-OSI Global: Lab-grown mini brains: we can’t dismiss the possibility that they could one day outsmart us, WebNews- English, (October 25, 2019 Friday)
57. 'Mini brain' is developed from human stem cells; 'Mini brain' is developed from human stem cells, Hannah Devlin, The Times (London), NEWS; FRONT PAGE; Pg. 1,4, (August 29, 2013 Thursday)
58. Mini-brains fire up like the real thing, Ruby Prosser Scully, New Scientist, NEWS; News & Technology; Pg. 10, (July 3, 2019)
59. Mini-brains grown from stem cells don't think, but they do show 'complex' neural activity, researchers say, By Susan Scutti, CNN, CNN Wire, (June 27, 2019 Thursday)
60. 'Mini-brain' made from stem cells; Miniature brains have been created in test tubes by stem cell scientists who claim they could help combat inherited neurological disorders., By Nick Collins Science Correspondent, telegraph.co.uk, NEWS, (August 28, 2013 Wednesday)
61. Minibrains, morality, and mimicking the human conscience , Katherine Lee, The Daily Campus: University of Connecticut, OPINION; Pg. 1, (September 6, 2019 Friday)
62. Mini-brains raise big hopes and fears; Synthetic neuroscience will soon face serious ethical issues, Financial Times (London, England), LEADER; Pg. 8, (August 31, 2013 Saturday)
63. Mini-brains revisited; the power of infographics; and future-focussed decision making, ABC Transcripts (Australia), (December 7, 2016 Wednesday)
64. MINI-ORGANS: NEXT-GEN LAB MODEL, NOT THE CHILD OF FRANKENSTEIN, States News Service, States News Service, (October 25, 2018 Thursday)
65. Mini-organs, sex in aged care and mental health at the GP's office, ABC Transcripts (Australia), (September 23, 2019 Monday)
66. 'MINI-PLACENTAS' COULD PROVIDE A MODEL FOR EARLY PREGNANCY, States News Service, States News Service, (November 28, 2018 Wednesday)
67. MOUNTING BRAIN ORGANOID RESEARCH REIGNITES ETHICAL DEBATE PENN NEUROSCIENTISTS CALL FOR ETHICAL FRAMEWORK GROUNDED IN SCIENTIFIC PRINCIPLES FOR TRANSPLANTING HUMAN "MINI-BRAINS" INTO ANIMALS AS FIELD EVOLVES, States News Service, States News Service, (October 3, 2019 Thursday)
68. Nature Communications Journal Issues 362 Research Articles in March 2019 Edition, Targeted News Service, Targeted News Service, (April 5, 2019 Friday)
69. NEUROSCIENTISTS GAIN NEW INSIGHTS THROUGH INNOVATION, States News Service, States News Service, (November 1, 2018 Thursday)
70. Neuroscientists have created 'mini brains' from human tissue that can FEEL and even suffer, say experts, as they warn an 'ethical line may have been crossed', Ian Randall For Mailonline, MailOnline, SCIENCE; Version:1, (October 21, 2019 Monday)
71. Neurosurgery Journal Issues 423 Research Articles in September 2019 Supplemental Edition, Targeted News Service, Targeted News Service, (August 21, 2019 Wednesday)
72. New lab-grown brains are as active as premature babies' - but scientists promise they can't 'think', Natalie Rahhal Deputy Health Editor For Dailymail.com, MailOnline, HEALTH; Version:1, (August 29, 2019 Thursday)
73. Nobel factory Cambridge lab prospers with stable funding, Financial Times (London, England), BUSINESS; Pg. 2, (December 10, 2018 Monday)
74. Olfactory neuroblastoma: A clinicopathological experience of a rare entity from Pakistan, Saroona Haroon, Muhammad Usman Tariq, Aisha Memon, Saira Fatima and Sheema Habibul Hasan, Pakistan Journal of Medical Sciences, (June 30, 2016 Thursday)
75. Organoids Are Not Brains. How Are They Making Brain Waves?; matter, Carl Zimmer, The New York Times , SCIENCE, (August 29, 2019 Thursday)
76. Pea brains haven't a thought in their heads. Yet; Growing new body parts could have enormous benefits - but it reopens old arguments about what makes us human, Colin Blakemore, The Times (London), EDITORIAL; OPINION, COLUMNS; Pg. 19, (August 31, 2013 Saturday)
77. Perelman School of Medicine: Mounting Brain Organoid Research Reignites Ethical Debate, Targeted News Service, Targeted News Service, (October 4, 2019 Friday)
78. venkat reddy. (September 5, 2019 Thursday). Personalized Medicine Market to Witness Disruption by Path-breaking Innovations Curing Glioblastoma and Pediatric Cancer Leveraging Artificial Intelligence. Newstex Blogs MarketersMedia News Hub. Retrieved from https://advance-lexis-com.prox.lib.ncsu.edu/api/document?collection=news&id=urn:contentItem:5X04-F101-F03R-N4VB-00000-00&context=1516831.
79. Targeted News Service. (February 4, 2019 Monday). PLOS Computational Biology Journal Issues 40 Research Articles in January 2019 Edition. Targeted News Service. Retrieved from https://advance-lexis-com.prox.lib.ncsu.edu/api/document?collection=news&id=urn:contentItem:5VBP-HBW1-DYG2-R0D6-00000-00&context=1516831.
80. Professor researches ethical growth, use of human brain organoids, Nathan Lesch, The Observer: Case Western Reserve University, NEWS; Pg. 1, (January 25, 2019 Friday)
81. Progress with lab-grown 'minibrains' sparks ethical debate in scientific community; Scientists and ethicists call for public discussion on responsibilities for brain cell researchers, Carolyn Y Johnson, The Independent (United Kingdom), SCIENCE; Version:1, (September 3, 2018 Monday)
82. RAT'S CREEPY Scientists give rats consciousness by implanting tiny HUMAN brains inside them; Stanford University bioethicist says as the lab rats become more human-like they may one day be 'entitled to some kind of respect', By Margi Murphy, thesun.co.uk, SCIENCE; Version:2, (November 8, 2017 Wednesday)
83. Rats with HUMAN brains - human/rodent hybrids created in lab spark ethical nightmare, Lara Deauville, Express Online, (November 8, 2017 Wednesday)
84. REGENERATION MECHANISM DISCOVERED IN MICE COULD PROVIDE TARGET FOR DRUGS TO COMBAT CHRONIC LIVER DISEASE, States News Service, States News Service, (November 4, 2019 Monday)
85. Register of Commission documents: more technologies which could change our lives Document date: 2017-07-14 EPRS_IDA(2017)598626 In-Depth Analysis, European Union News, (September 12, 2017 Tuesday)
86. REP. MARSHA BLACKBURN HOLDS A HEARING ON BIOETHICS AND FETAL TISSUE, CQ Transcriptions, (March 2, 2016 Wednesday)
87. Maggie Lynch, . (November 26, 2018 Monday). Repositive expands preclinical cancer model offering. Outsourcing-Pharma.com. Retrieved from https://advance-lexis-com.prox.lib.ncsu.edu/api/document?collection=news&id=urn:contentItem:5TTY-6MC1-JC6M-X3SJ-00000-00&context=1516831.
88. Researchers grow brain parts to study development, disease, By MALCOLM RITTER, Associated Press Online, DOMESTIC NEWS, (November 5, 2015 Thursday)
89. Rodents With Part-Human Brains Pose A New Challenge For Bioethics, WebNews- English, (October 4, 2019 Friday)
90. SANGER INSTITUTE CANCER RESEARCHER'S INNOVATION RECOGNISED, States News Service, States News Service, (October 8, 2019 Tuesday)
91. (November 17, 2017 Friday). Schizophrenia originates early in pregnancy, 'mini-brain' research suggests. US Official News. Retrieved from https://advance-lexis-com.prox.lib.ncsu.edu/api/document?collection=news&id=urn:contentItem:5R07-82V1-J9XT-P3NT-00000-00&context=1516831.
92. Scientists grow first 'brain' from human stem cells, Hannah Devlin, Science Editor, thetimes.co.uk, SCIENCE, (August 28, 2013 Wednesday)
93. SCIENTIST: MOST COMPLETE HUMAN BRAIN MODEL TO DATE IS A 'BRAIN CHANGER', US Fed News, (August 18, 2015 Tuesday)
94. Scientists Are Giving Dead Brains New Life. What Could Go Wrong?; Feature, Matthew Shaer, The New York Times , MAGAZINE, (July 2, 2019 Tuesday)
95. SCIENTISTS CREATE BRAIN-LIKE BLOBS IN TEST TUBES, States News Service, States News Service, (August 28, 2013 Wednesday)
96. Scientists create human 'mini-brain', Clive Cookson, Science Editor, FT.com, (August 28, 2013 Wednesday)
97. Scientists develop fast cheap method for growing 'brains-in-a-dish', Iran Daily, (September 8, 2018 Saturday)
98. SCIENTISTS ENGINEER GENE PATHWAY TO GROW BRAIN ORGANOIDS WITH SURFACE FOLDING, States News Service, States News Service, (December 29, 2016 Thursday)
99. Scientists implant tiny human brains inside rats to give them 'enhanced intelligence' provoking an ethical outcry, HARRY PETTIT FOR MAILONLINE, MailOnline, SCIENCE; Version:4, (November 8, 2017 Wednesday)
100. Scientists 'may have crossed ethical line' in growing human brains, Yerepouni Daily News, (October 21, 2019 Monday)
101. Scientists: Mini-brains a delicate discussion Organoids important for research, but potential moral status must be handled transparently, Bradley J. Fikes, The San Diego Union Tribune, BUSINESS; C; Pg. 1, (October 5, 2019 Saturday)
102. Scientists observe neural activity in lab-grown mini-BRAINS (but insist the simplified organs can't 'think' for themselves), Cheyenne Macdonald For Dailymail.com, MailOnline, SCIENCE; Version:1, (June 27, 2019 Thursday)
103. Scientists See Human-Like Brain Waves in Lab-Grown Mini-Brains, WebNews- English, (August 29, 2019 Thursday)
104. Scientists to grow 'mini-brains' using Neanderthal DNA; Geneticists hope comparing prehistoric and modern biology will help them understand what makes humans unique, Hannah Devlin in Leipzig, The Guardian(London), SCIENCE; Version:1, (May 11, 2018 Friday)
105. Screens spoiling brains?, ABC Transcripts (Australia), (November 23, 2018 Friday)
106. SEN. LAMAR ALEXANDER HOLDS A HEARING ON CONTINUING AMERICA'S LEADERSHIP IN MEDICAL INNOVATION FOR PATIENTS, CQ Transcriptions, (March 10, 2015 Tuesday)
107. Society for Neuroscience: Better Way to Transplant Human Stem Cells, Targeted News Service, Targeted News Service, (November 13, 2018 Tuesday)
108. Sowing Stem Cells: Lab-Grown Organoids Hold Promise for Patient Treatments, Targeted News Service, Targeted News Service, (June 7, 2017 Wednesday)
109. Spanish scientists create human and monkey chimeras in China, CE Noticias Financieras English, (July 30, 2019 Tuesday)
110. 'Synthetic' human embryos are on the horizon, but ethics surrounding them are complicated, Postmedia Breaking News, HEALTH, (April 29, 2018 Sunday)
111. The Real Story About Organoids: What You Should Know About “Brains In A Dish”; WebNews- Academic, (October 27, 2019 Saturday)
112. The reanimators: Giving dead pig brains new life, Irish Examiner, VIEWS NEWS, (July 13, 2019 Saturday)
113. The scientist who grows tiny brains in her laboratory; Dr Madeline Lancaster has discovered how to turn stem cells into grey matter. It could lead to treatments for autism and spinal injury, Simon Crompton, thetimes.co.uk, TIMES2; Version:1, (October 7, 2019 Monday)
114. These mice have brains that are part human. So are they mice, or men?, By Bianca Nogrady, ABC Premium News (Australia), (July 29, 2018 Sunday)
115. Thinking ahead, Alex Pearlman, New Scientist, NEWS; Comment; Pg. 26-27, (May 2, 2018)
116. Tiny Lab-Grown 'Brains' Raise Big Ethical Questions, NPR All Things Considered, (April 25, 2018 Wednesday)
117. UCLA In the News January 3, 2019, US Official News, Plus Media Solutions, (January 3, 2019 Thursday)
118. UCSD Researchers Detail Simpler Way To Develop Miniaturized Human Brains, City News Service, (September 6, 2018 Thursday)
119. University of California-San Diego: Machine Learning Algorithm Can't Distinguish These Lab Mini-Brains From Preemie Babies, Targeted News Service, Targeted News Service, (August 30, 2019 Friday)
120. University of Pennsylvania: Mounting Brain Organoid Research Reignites Ethical Debate, Targeted News Service, Targeted News Service, (October 13, 2019 Sunday)
121. Wary Hopes for Lab's Cerebral Cells, By CARL ZIMMER, The New York Times, Section D; Column 0; Science Desk; Pg. 1, (September 3, 2019 Tuesday)
122. We Are ‘Perilously Close’ to Creating Sentient Mini-Brains in a Dish, Experts Warn – ScienceAlert, WebNews- English, (October 22, 2019 Tuesday)
123. What are organoids?: A Mint on Sunday audio story, MINT, (September 2, 2017 Saturday)
124. What's Wrong With Growing Blobs of Brain Tissue?, Ed Yong, Atlantic Online, (April 25, 2018 Wednesday)
125. WHO IS HARVARD GENETICIST GEORGE CHURCH?, HARRY PETTIT FOR MAILONLINE, MailOnline, SCIENCE; Version:2, (July 3, 2018 Tuesday)
126. World first: Human-level brain waves detected in lab-grown mini-brains, MarketLine NewsWire (Formerly Datamonitor), TECHNOLOGY & TELECOMMUNICATIONS, (August 29, 2019 Thursday)
127. Lab-grown mini brains could soon outsmart us, The Conversation, Newstex Blogs  TheNextWeb.com, (December 27, 2019 Friday)
128. Lab-grown mini brains could soon outsmart us, IT Next, (December 27, 2019)
129. New Cyborg Technology May Reverse Paralysis And Replace Functionality, Newstex Blogs  International Business Times News, (December 11, 2019 Wednesday)
130. A highly subjective list of some of this year's notable young startups, Greentechlead.com, (December 26, 2019 Thursday)

## 
